# Sinonasal adenoid cystic carcinoma-role of on-site FNAC: a case report

**DOI:** 10.1186/s12901-018-0053-4

**Published:** 2018-05-09

**Authors:** Santosh Tummidi, Kanchan Kothari, Roshni Patil, Shruti S. Singhal, Vyoma Shah

**Affiliations:** Department of Pathology, Seth GSMC & KEMH, Parel, Mumbai, Maharashtra 4900012 India

**Keywords:** Sinonasal, Adenoid cystic carcinoma, C-kit, Fine needle aspiration, Toluidine blue

## Abstract

**Background:**

Adenoid cystic carcinoma (ACC), a rare tumor of epithelial cell origin, commonly arises from the major salivary glands. Uncommonly it may be found outside the salivary glands and it's especially rare in the nasal cavity.

**Case presentation:**

A 71-year female had complaints of swelling at the base of nose, Fine needle aspiration (FNA) from the swelling revealed features of adenoid cystic carcinoma; cell block & IHC for CD-117 was positive.

**Conclusions:**

Sino-nasal ACC (SNACC) continues to pose diagnostic and therapeutic challenges to clinicians. Due to its rarity & vague presentation, early diagnosis requires a high index of suspicion. FNA can be used as an invaluable diagnostic tool in the evaluation of these lesions. Since it’s incidence in sinonasal region is rare; our attempt to report this case will heighten the physician’s awareness of this disease, helping further treatment.

## Background

Adenoid cystic carcinoma is a rare malignant tumour, accounting for 1-2% of all sinonasal malignant tumours & representing < 0.15% of all malignant head and neck tumours, regardless of site and histology [1, 2].

SNACC is locally destructive and can show perineural/perivascular spread, resulting in a high rate of recurrence despite aggressive surgical resection, sometimes as late as 10-20 years after initial management [3]. FNA can be done form any accessible site in the head and neck region. It is an accurate and cost-effective process with a quick turn-around time. We found limited studies in literature regarding role of FNA in the diagnosis of sinonasal tract neoplasms [2–4]. Cytological diagnosis of SNACC can help in early diagnosis, treatment and prevention of recurrence. We report a case of ACC of nasal cavity and paranasal sinus in a 71-year-old lady diagnosed on cytology.

## Case presentation

**Fig. 1:**
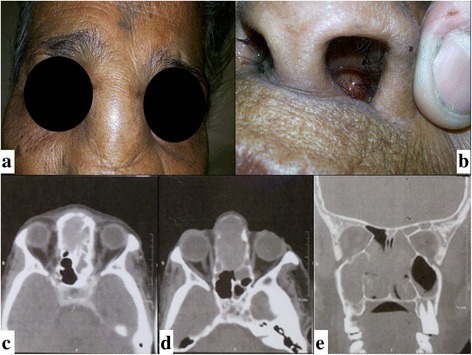
**a**, **b** O/E a diffuse swelling over the root of nose, measuring approx. 2 × 2 cm, hard and cystic in feel, non-tender, overlying skin was pinchable; **c**, **d**, **e** MDCT scan of paranasal sinuses showed a large mass lesion involving the nasal cavity with bony destruction of nasal septum, thinning of nasal turbinate’s, deossification of hard palate, nasal bones &medial wall of the maxillary sinuses

A 71-year female, presented to our outpatient department with complaints of swelling over dorsum of nose since 4-5 months. She had a history of fall with sudden increase in size in the last 2 months. There were no ocular complaints i.e. diplopia, discharge, redness or itching in her eyes. She was a known case of hypertension under treatment since 2 years. ENT examination revealed absence of air blast on both sides. External examination showed a small cystic swelling over root of nose extending to mid portion of nose (Fig. 1a, b). Anterior rhinoscopy revealed a mass occluding both nasal cavities, with mild maxillary and ethmoid tenderness. CT scan of paranasal sinuses revealed a moderately large mass lesion involving the nasal cavity with bony destruction of nasal septum, multiple inter-ethmoid bony septae and nasal turbinates with bony thinning and deossification of a part of the hard palate, nasal bones and medial wall of the maxillary sinuses. The mass measured approximately 5.7 × 4.7 × 3.3 cm (Fig. 1c, d, e). A small component of this lesion was entering the subcutaneous tissue on the left side of the nasal bridge through a defect in the bone, for which she was referred for fine needle aspiration.

**Fig. 2:**
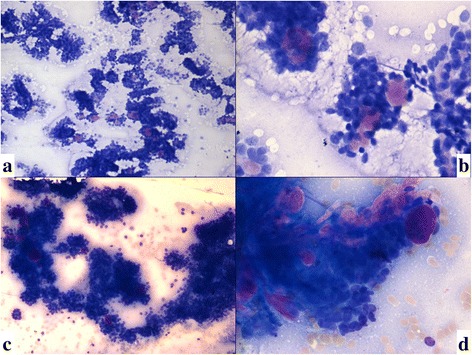
**a**, **b**, **c**, **d** Cytosmears were very cellular with clusters and papillaroid fragments of basaloid looking cells. Seen admist were tumor cells are rounded globules & islands of uniform hyaline material that stained magenta. (Toluidine blue, × 10, 40; Geimsa, × 10,× 40)

**Fig. 3:**
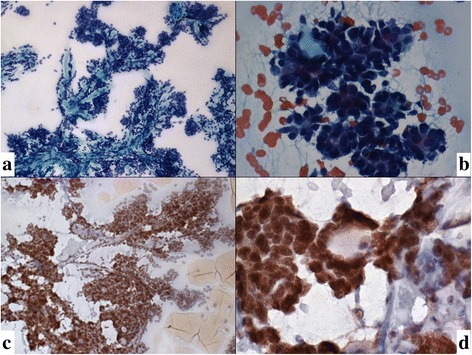
**a**, **b** Cytosmears with clusters and papillaroid fragments of basaloid looking cells and transparent homogenous looking hyaline globules.(Pap, × 10,× 40); (**c, d**): Immunocytochemistry of the acetone fixed slide was strong positive for c-Kit(IHC,× 10,× 40)

**Fig. 4:**
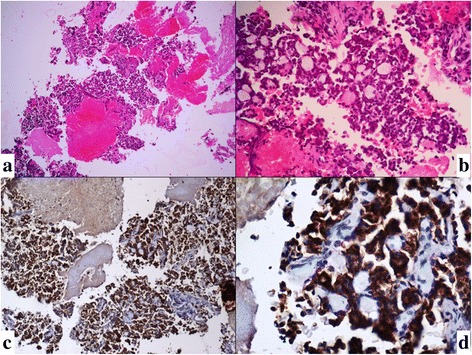
**a**, **b** Cell block preparation showed classical cribriform pattern of ACC with IHC positivity for c-Kit (**c**, **d**) (H&E, × 10,× 40)

FNA was done using 23-gauge needle and smears were stained with PAP, Geimsa & toluidine blue. Cytosmears were very cellular with clusters of cells which were small, basaloid with hyperchromatic nuclei & very scant cytoplasm. Nuclear moulding was seen at many places. Seen amidst the tumor cells were rounded globules and finger like projections of uniform hyaline material that stained magenta on Geimsa. Occasional clusters of histiocytes were seen. A few papillaroid fragments with central fibro vascular core were also noted (Figs. 2 and 3). A cell block was made and it revealed a cribriform pattern with basaloid cells rimming hyaline globules. C-kit IHC was done on the cell block material and it showed strong and diffuse positivity (Figs. 3c, d and 4) Thus, a diagnosis of a sinonasal malignant salivary gland type neoplasm - adenoid cystic carcinoma was given.

## Discussion

ACC is the 3rd commonest sinonasal malignancy. These tumors pose challenges in terms of their management because of the difficulty in accessing the tumor, slow progression, local recurrence, metastases and low radio-curability. Thus early diagnosis and treatment will allow better outcomes in terms of reduced morbidity and risk of residual or recurrence of disease [5, 6].

The most common age group for these tumors is 40-60 years with a female predilection. Maxillary sinus followed by nasal cavity is the most commonly affected site [7]. Symptoms can include nasal obstruction, difficulty in breathing, pain (head paranasal, orbital, auditory, dental), epistaxis, enlarging mass/swelling, bone destruction, nasal discharge, facial anesthesia, blurred vision, exophthalmos, facial paralysis and cognitive deficits [3, 6].

Cytological diagnosis of ACC: The typical morphology is the presence of hyaline globules (basement-membrane like material) surrounded by tumor cells. Finger-like or rounded material is seen between cell clusters, the cells show uniform round to oval hyperchromatic nuclei and little cytoplasm [8, 9].

IHC reveals that the luminal tumor cells are diffusely positive for cytokeratin, EMA, CEA and CD-117 (c-Kit) indicating ductal origin. Those that surround the pseudo cysts show S-100, SMA, Calponin positivity and variable cytokeratin positivity suggesting a myoepithelial cell differentiation. Strong c-KIT expression will be seen in almost all neoplastic cells in the solid pattern, all cells surrounding pseudocysts in the cribriform pattern, and all luminal cells in the tubular pattern. Over expression of p53 and Ki-67 with loss of myoepithelial markers is found in high-grade tumors. Affinity of tumor cells to proliferate along the basement membranes is responsible for frequent invasion of tumor cells into basement membrane-rich tissues i.e. peripheral nerves, blood vessels and skeletal muscles. Cytogenetically, the most consistent although not exclusive reported alterations have been at chromosomes 6q, 9p and 17p12-13 regions [10].

The differentials of SNACC includes basaloid squamous cell carcinoma (BSCC), PLGA, olfactory neuroblastoma(ONB), neuroendocrine carcinoma (small cell) [11]. BSCC shows smaller, uniform but angulated nuclei [11]. PLGA usually shows round to oval nuclei, bland nuclear chromatin with moderate eosinophilic - clear cytoplasm, whereas SNACC shows more of basaloid features. IHC in PLGA shows positivity to cytokeratin, vimentin, S–100 protein, CEA [12]. ONB can present diagnostic difficulties with solid variant ACCs. Lobular pattern, with enlarged pleomorphic cells, hyperchromatic nuclei, variable nucleoli, raised mitosis/ necrosis (confluent areas or individual cell) & true rosettes (Flexner-Wintersteiner type) may be seen along with IHC reactivity for chromogranin, synaptophysin and CD56 in ONB [11].

Sinonasal undifferentiated carcinoma (SNUC) is another aggressive malignant neoplasm of the nasal cavity/ paranasal sinuses with no definite histologic differentiation. Tumor cells show marked pleomorphism, hyperchromasia, prominent nucleoli, prominent mitotic activity/ necrosis but CD117 and p63 negative in SNUC [13, 14].

Neuroendocrine carcinoma (small cell/ large cell) are clinically more destructive, show high nuclear to cytoplasmic ratio, salt pepper chromatin, high mitotic rate and tumor necrosis along with IHC showing dot-like to punctate keratin immunoreactivity and strong, diffuse synaptophysin, chromogranin, CD56, and NSE reactivity. To note, CD117 is positive in both tumors, while CK7 is negative in NEC and strongly positive in most SNACC [15].

ACC is a radiosensitive tumor; however, in most patients, radiation is not the only cure. Advantage of using postoperative radiation therapy is to clear positive margins that are left after surgery. Despite radiation, in patients who had undergone surgery with postoperative radiation, the recurrence rate was high (65%). The possible reason being perineural spread of ACC. Few authors have postulated that postoperative radiation delays rather than preventing recurrences. Overall 5-year survival rate in SNACCs ranges from 50 to 86% and overall recurrence rate being 56% [6].

## Conclusion

SNACC tumours are rare. Cytology along with ICC can help in early diagnosis and treatment of SNACC, especially in tumors which have extended to the soft tissue thus making them amenable to aspiration. Biopsy can play a role in cases where FNAC material is limited or non-accessible sites. Surgery in combination with postoperative radiation provides better overall survival rate. Chemotherapy still has not been proven effective in the treatment of this disease.

## Abbreviations

ACC: Adenoid cystic carcinoma
ASC: Adenosquamous carcinoma
BSCC: Basaloid squamous cell carcinoma
CD: Clusters of differentiation
CEA: Carcino-embryonic antigen
CK: Cytokeratin
EMA: Epithelial membrane antigen
FNAC: Fine needle aspiration cytology
GFAP: Glial fibrillary acidic protein
ICC: Immunocytochemistry
IHC: Immunohistochemistry
MDCT: Multiple detector computed tomography
ONB: Olfactory neuroblastoma
PLGA: Polymorphous low-grade adenocarcinoma
SNACC: Sino-nasal ACC
SNUC: Sinonasal undifferentiated carcinoma
